# Cryo‐kinetics Reveal Dynamic Effects on the Chemistry of Human Dihydrofolate Reductase

**DOI:** 10.1002/cbic.202100017

**Published:** 2021-05-04

**Authors:** Aduragbemi S. Adesina, Louis Y. P. Luk, Rudolf K. Allemann

**Affiliations:** ^1^ School of Chemistry Cardiff University Park Place Cardiff CF10 3AT UK

**Keywords:** cryo-kinetics, DHFRs and protein motions, heavy enzyme, human dihydrofolate reductase

## Abstract

Effects of isotopic substitution on the rate constants of human dihydrofolate reductase (HsDHFR), an important target for anti‐cancer drugs, have not previously been characterized due to its complex fast kinetics. Here, we report the results of cryo‐measurements of the kinetics of the HsDHFR catalyzed reaction and the effects of protein motion on catalysis. Isotopic enzyme labeling revealed an enzyme KIE (*k*_H_^LE^/*k*_H_^HE^) close to unity above 0 °C; however, the enzyme KIE was increased to 1.72±0.15 at −20 °C, indicating that the coupling of protein motions to the chemical step is minimized under optimal conditions but enhanced at non‐physiological temperatures. The presented cryogenic approach provides an opportunity to probe the kinetics of mammalian DHFRs, thereby laying the foundation for characterizing their transition state structure.

## Graphical abstract

**The results of cryo‐measurements** of the kinetics of the human dihydrofolate reductase (HsDHFR) catalyzed reaction and the effects of protein motion on catalysis are reported. Isotopic enzyme labeling revealed an enzyme KIE (k_H_^LE^/k_H_^HE^) close to unity above 0 °C; however, the enzyme KIE was increased to 1.72±0.15 at −20 °C, indicating that the coupling of protein motions to the chemical step is minimized under optimal conditions but enhanced at non‐physiological temperatures.

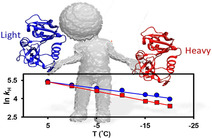

Enzymes are recognized for both their catalytic efficiency and dynamic nature. Protein motions on the timescale of bond vibrations (femtoseconds) have been proposed to facilitate catalysis by coupling to the chemical reaction.^[1]^ However, the physical importance of such dynamic coupling remains unclear.[^1a^, ^1d^, ^2^] Measurements of kinetic isotope effects (KIEs) can shed light on how changes in protein dynamics from isotopic substitution effect enzyme catalysis. KIEs obtained when isotopes (^1^H, ^12^C and ^14^N) of a substrate or an enzyme are replaced with their heavy counterparts (^2^H, ^13^C and ^15^N) report on bonding and geometry differences between reactants and the transition state.^[3]^ However, for enzymatic reactions, the measurement of KIEs is often difficult and the real KIE value can become masked when the elementary step of the reaction is fast or the observed rate constant reflects multiple kinetic steps.^[4]^

Dihydrofolate reductase (DHFR) catalyzes the transfer of the *pro*‐R hydride from C4 of NADPH to the C6 of 7,8‐dihydrofolate (DHF) to produce tetrahydrofolate (THF) (Figure 1A). This reaction is part of the biosynthesis of several amino acids, purines and thymidylate. DHFR is also a key target for drugs such as the anticancer agent methotrexate, and the antibiotic trimethoprim. Many previous investigations of DHFR catalysis have focused on bacterial enzymes, especially DHFR from *E. coli* (EcDHFR) because this enzyme undergoes significant conformational changes during catalysis (Figure 1B).^[5]^ Kinetic analysis of the reaction catalyzed by EcDHFR labeled with heavier isotopes revealed that coupling of fast protein dynamics to the chemical coordinate is minimal;^[6]^ indeed such coupling induces recrossing of the transition state back to the reactant state and is hence unfavorable for progression to products.[^6a^, ^6b^] This finding was supported in the investigations of DHFR homologs from hosts adapted to thrive at wide range of temperatures ranging from 2 to 80 °C.[^2c^, ^7^]

**Figure 1:**
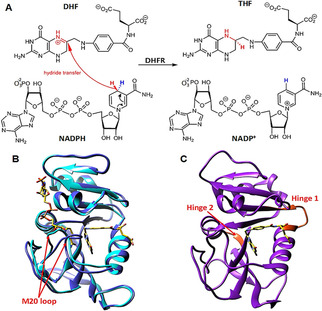
(A) Reaction catalyzed by dihydrofolate reductase. (B) Overlay of cartoon representations of EcDHFR showing active site loop (M20) in the closed (cyan, PDB 1RX2) and occluded conformation (blue, PDB 1RX6). (C) Cartoon representation of HsDHFR (purple, PDB 1DHF) with hinge 1 (residues 39–49) and hinge 2 (residues 129–131) proposed to be important for ligand flux (orange).^[8]^ The ligands, folate and NADP^+^ are shown as sticks (yellow).

Although the tertiary structure of DHFRs from bacteria and mammals are conserved, significant biophysical differences exist between them.^[8]^ For example, the large scale conformational movements of EcDHFR during catalysis^[5]^ are not seen in human DHFR (HsDHFR), but instead slight twists of residues at the hinge of the cofactor binding domain control ligand binding (Figure 1C).[^8^, ^9^] In addition, trimethoprim binds selectively to bacterial DHFRs.^[10]^ These observations suggest that the role of protein motions on the chemical step during HsDHFR catalysis cannot be inferred from investigations of its bacterial homologs. Previously, a theoretical analysis of the transition state of HsDHFR suggested that fast protein vibrations couple to the chemical step of the reaction,^[1b]^ but this proposition has been disputed by others.^[11]^ However, a lack of good experimental data due to the very fast reaction and complex kinetics of the HsDHFR catalyzed reaction[^4b^, ^11b^, ^12^] have so far prevented the experimental verification of the computational proposal.

Here we report cryo‐kinetic measurements between −20 and +5 °C to probe the effects of substrate and enzyme isotopic substitution on the rate constant of HsDHFR. Our results indicate that fast protein dynamics do not couple to the chemical step and are indeed minimized at physiological temperatures.

While in bacterial DHFRs hydride transfer becomes rate‐limiting above pH 8.5 under steady state conditions, the chemical step during catalysis by mammalian DHFRs is rate‐limiting only above pH 9.5.^[4b]^ The rate constant for the HsDHFR‐catalyzed H‐transfer was measured at pH 10.0 under steady‐state conditions to reduce limitations associated with the fast and complex rate reported previously.[^4b^, ^11b^, ^12^] The rate constant (*k*_cat_) obtained for the transfer of hydride from NADPH to DHF at 20 °C was 1.58±0.01 s^−1^; it reduced two‐fold when (*R*)‐[4‐^2^H]‐NADPH (NADPD) was used resulting in a substrate *k*_cat_^H^/*k*_cat_^D^ of 2.0±0.1. Further measurements between 5 and 35 °C gave linear Arrhenius plots for *k*_cat_^H^ and *k*_cat_^D^ and a moderate temperature dependent KIE_cat_ (Figure 2 and Table S1). The KIE_cat_ values reported here are identical to those derived from competitive measurements,^[11b]^ and slightly larger than the ones measured previously under similar non‐competitive conditions,^[4b]^ most likely as a consequence of the minor difference in the reaction conditions (150 mM KCl vs 100 mM NaCl).^[4b]^ The ionic strength has been shown to affect the kinetics of the DHFR catalyzed reaction.^[13]^

**Figure 2:**
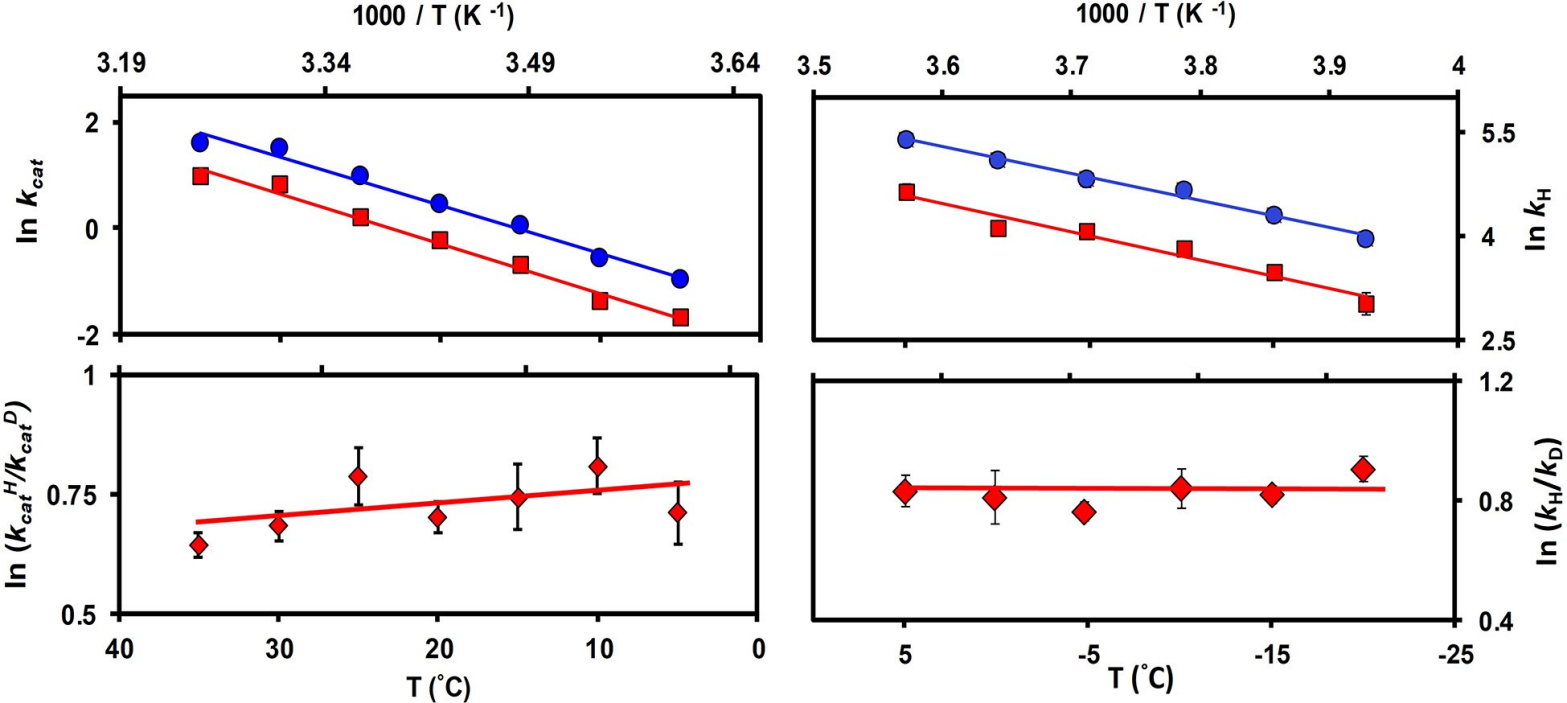
(Top) Arrhenius plots for hydride (•) and deuteride (▪) transfer and (Bottom) the corresponding H/D substrate KIEs (♦). (Left) Steady‐state rate (*k*_cat_) plots at pH 10.0 measured between 5 and 35 °C and (**Right**) pre‐steady‐state rate (*k*_H/D_) plots at pH 8.5 measured in the presence of 30 % methanol between −20 and +5 °C.

Despite the significance of our measured KIE_cat_ values, complex multiple time‐course kinetics were observed at lower temperatures (Figure S1 and Supporting Information for details) suggesting that the chemical step may be slightly masked by other steps. This agrees with previous investigations that suggested that HsDHFR catalysis exhibits kinetic complexity under various analytical conditions.[^4b^, ^12b^]

Although pre‐steady state kinetics offer a more direct approach to investigate transient reactions,^[14]^ previous attempts to measure pre‐steady state rate constants for the HsDHFR‐catalyzed reaction under physiological conditions were unsuccessful because hydride transfer occurs within the dead time of standard stopped‐flow instruments.[^4b^, ^11b^, ^12^] We therefore explored whether measurement of pre‐steady state kinetics at sub‐zero temperatures might allow the measurement of KIEs due to the reduced reaction rate. To this end, we modified a conventional stopped‐flow instrument to create a cryo‐kinetic setup to measure the hydride transfer rate of the reaction and its temperature‐dependence between −20 °C and +5 °C (Figure 3). 30 % methanol was used as a cosolvent and the pH adjusted to 8.5 to further reduce the transient rate. The choice of methanol is based on previous work on two enzymes where Arrhenius plots obtained in the presence of methanol at sub‐zero temperatures were linear and parallel to those measured above 0 °C without the cosolvent.^[15]^ Since the apparent *pK*_a_ of HsDHFR reaction is unchanged between pH 5.0 and 9.0,^[4b]^ rate constants measured at pH 8.5 reflect physiological conditions where the hydride is transferred to mostly protonated substrates.^[16]^

**Figure 3:**
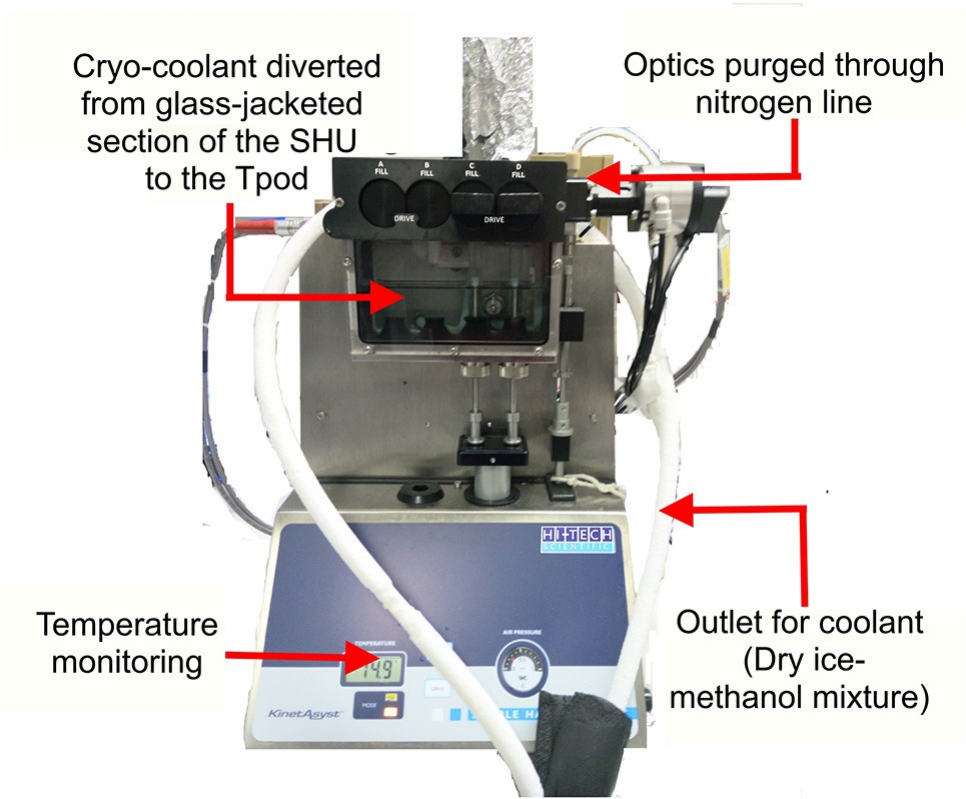
TgK stopped‐flow Sample Handling Unit (SHU) showing cryogenic modifications. The TPod is an attachment that fits between the SHU and the cell block, allowing the cell block to be thermostated separately.

The pre‐steady state rate constants at −20 °C and pH 8.5 were 51.4±2.4 s^−1^ and 20.8±3.5 s^−1^ for NADPH and NADPD, respectively, resulting in a substrate *k*_H_/*k*_D_ of 2.5±0.1. The KIEs_H/D_ were largely temperature independent between −20 to +5 °C (Figure 2 and Table S2). The rate constant measured under pre‐steady state at pH 8.5 was significantly higher than that obtained under steady state conditions at pH 10.0 (even at similar temperatures). The activation energies of the reactions were also different, 7.8±0.4 and 15.5±0.7 kcal ⋅ mol^−1^ for pre‐steady and steady state, respectively, even though the KIEs_H/D_ for both conditions were identical. A similar observation was reported for EcDHFR at pH 7.0 under pre‐steady state and at pH 9.5 under steady state, where a 200‐folds difference in reaction rate constants and two‐fold difference in activation energies were observed^[6a]^ despite KIEs_H/D_ being identical under both conditions.^[17]^ Theoretical analysis revealed that such discrepancy can arise from the difference in activation barrier recrossing manifested in altered transmission coefficients.^[6a]^

To investigate the influence of methanol on the reaction, while avoiding the fast and complex kinetics of the enzyme, we employed NADPD to determine the reaction kinetics above 0 °C. The pre‐steady state rate constant (*k*_D_) in the absence of methanol at 0 °C was 97.9±3.1 s^−1^ but this reduced to 71.9±3.25 when 30 % methanol was present. Similarly, rate constants *k*_D_ measured between 0 °C and 20 °C were ∼25 % lower in 30 % methanol (Table S3). This agrees with earlier work that showed that the reduced rate in methanol correlates with a decrease in the dielectric constant of the buffer mixture.^[18]^ Our analysis revealed that the slope of the Arrhenius plots for *k*_D_ in the presence and absence of methanol were identical (Figure 4, Top), in agreement with previous results that indicated that methanol had a negligible effect on activation energies^[15]^ and the primary KIEs.[^13b^, ^18^, ^19^] The effect of methanol on the structural properties of HsDHFR was investigated. While the circular dichroism (CD) spectra indicated a minor structural change by the addition of the cosolvent (Table S4, Figure S4 and S5), the K_M_ values remained unchanged indicating that enzyme‐ligand interactions were not affected (Table S5). Although our previous work on the influence of cosolvents on the kinetics of DHFR from Thematoga maritima (TmDHFR) suggested that methanol, unlike other cosolvents, perturbs the enzyme structure, further investigation on monomeric DHFRs indicated that this effect relates specifically to the dimeric structure of the enzyme.[^18^, ^19^] In fact, the effect of methanol on DHFRs appears to be enzyme specific with the dimeric TmDHFR being the most structurally perturbed and the DHFR from the cold adapted Moritella profunda the least, even in the presence of 50 % methanol.^[19b]^ However, the addition of methanol reduced both the steady state (*k_cat_*) at pH 7.0 (Table S5) and the pre steady state rate constants (*k_H_*) above 10 °C.

**Figure 4:**
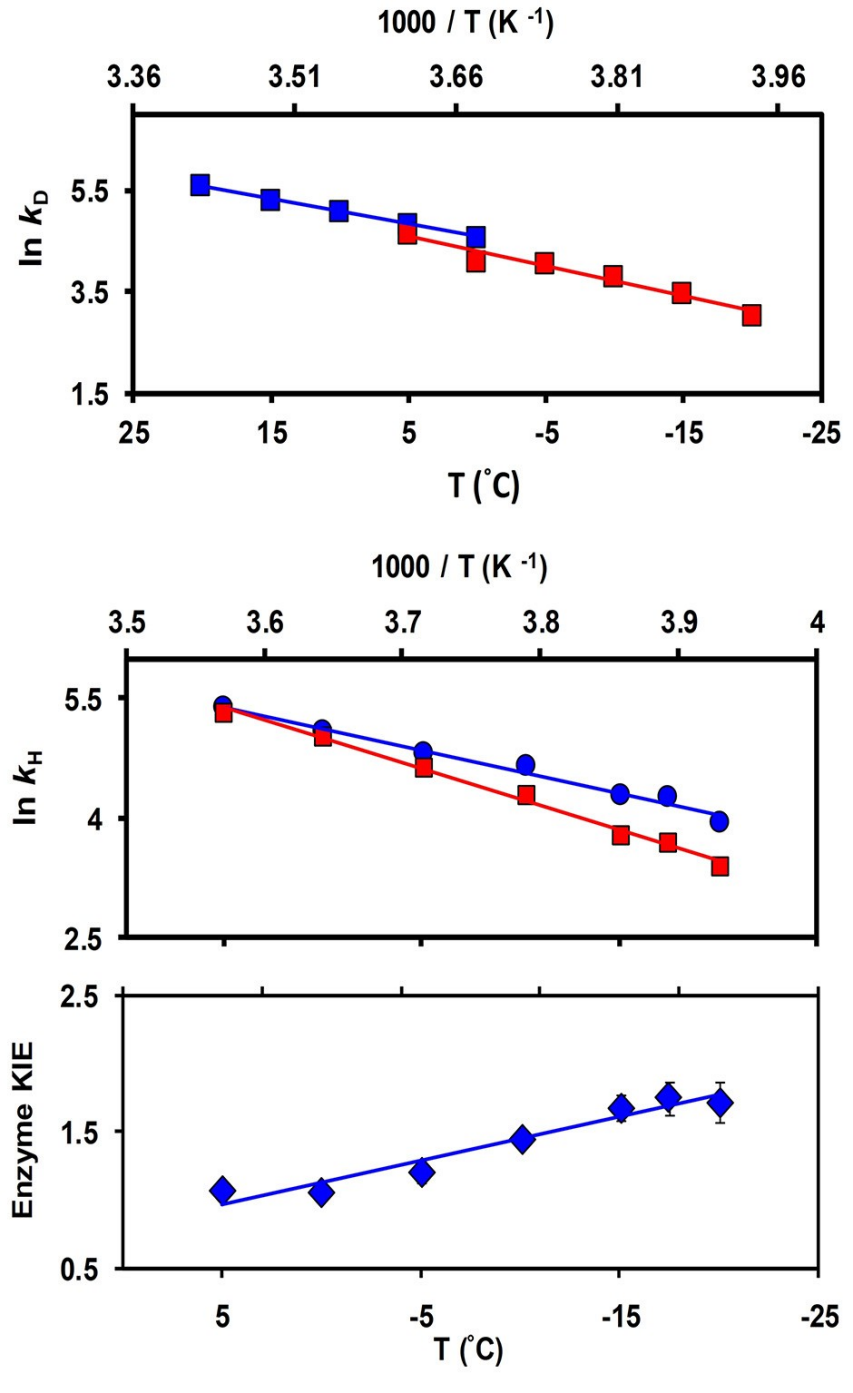
(**Top**) Arrhenius plots for deuteride transfer at pH 8.5 in the absence of 30 % methanol (▪) between 0 and +20, and in the presence of 30 % methanol (▪) between −20 and 0 °C. **(Middle)** Arrhenius plots of hydride transfer rate constants for light (•) and heavy (▪) HsDHFR at pH 8.5 and (**Bottom**) the corresponding enzyme KIEs (♦).

The dynamic behavior of HsDHFR was investigated by comparing the kinetic parameters of isotopically labeled (^13^C, ^15^N) “heavy” HsDHFR and the natural abundance “light” enzyme. Mass spectrometric analysis showed a mass increase of 5.1 % for the isotopically labeled enzyme (Figures S2 and S3). The CD spectra of light and heavy HsDHFR were indistinguishable, indicating that the isotopic substitution did not alter the secondary structure (Figures S4 and S5, Table S7). The steady‐state parameters *k*_cat_ and *K*_M_ (for NADPH and DHF at pH 7.0, 20 °C) of the light and heavy enzymes were identical within error (Table S6) agreeing with previous results that indicated that HsDHFR undergoes only minimal structural rearrangement during catalysis.^[8]^ Cryo‐kinetic measurement between −20 and +5 °C revealed that *k*_H_ of the light and heavy enzymes diverged significantly below 0 °C (Figure 4, Middle). The enzyme KIEs (*k*_H_^LE^/*k*_H_^HE^) increased from 1.07±0.05 at 5 °C to 1.72±0.15 at −20 °C (Figure 4, Bottom and Table S8) suggesting that the dynamic coupling of HsDHFR is significantly enhanced at sub‐zero temperatures. Similarly, for DHFR homologs from microbes adapted to temperatures from 2 and 80 °C, non‐unity enzyme KIEs were observed only under non‐physiological conditions.[^2c^, ^7^] Recent work has raised important questions on the proposed electrostatic similarity between enzyme isotopologues.^[21]^ An investigation of formate dehydrogenase suggested that isotopic labelling may induce a non‐mass dependent dynamic effect on the active site electrostatics, which is reflected in the differing values and slopes of H/T KIE of the isotopologues as well as their 2D infrared measurements.^[21b]^ However, an investigation of light and heavy HsDHFR indicated that the H/T KIEs of both isotopologues were identical at all temperatures investigated.^[11b]^

The KIEs_H/D_ of the heavy HsDHFR was measured at −15 and 5 °C and found to be virtually identical to that of the light enzyme (Table S11, Supporting Information). This examination suggests caution when comparing the substrate KIEs of the light and heavy enzymes.^[11b]^ As shown here, the rate constants of the individual enzymes show a clear effect caused by enzyme isotopic substitution, while substrate KIEs of the enzymes remained unperturbed even at sub‐zero temperatures.

Thermodynamic parameters provide additional insight into the transition state of chemical reactions. Indeed, the thermodynamic parameters of HsDHFR reveal that its activation free energy (ΔG^≠^) is distinctly lower than those observed for other DHFR homologs (Table 1). In addition, while both light and heavy HsDHFR have identical free activation energies (ΔG^≠^), their activation enthalpies (ΔH^≠^) and activation entropies (ΔS^≠^) were noticeably different (Table 1), aligning with a previous report that such observation arises from entropy‐enthalpy compensation.^[20b]^ Importantly, heavy HsDHFR exhibits a smaller ΔS^≠^ than the light enzyme, suggesting that the equilibrium reorganizational motions required by the enzyme to stabilize the transition state become hampered at lower temperatures. This observation is similarly reported for the heavy enzyme of the thermophilic DHFR from *Bacillus stearothermophilus* (BsDHFR) (Table 1).^[20b]^

**Table 1:** Activation parameters for hydride transfer reactions catalyzed by ‘light’ and ‘heavy’ DHFRs isolated from different species at pH 7.0 and 25 °C. HsDHFR parameters were determined at pH 8.5 and 0 °C.

	HsDHFR (Human, mesophilic)		EcDHFR^[a]^ (*E. coli*, mesophilic)		BsDHFR^[b]^ (moderately thermophilic)		MpDHFR^[c]^ (psychrophilic)	
	Light	Heavy	Light	Heavy	Light	Heavy	Light	Heavy
ΔS^≠^ (kcal mol^−1^ K^−1^)	−23±1	−11±1	−26±1	−30±2	−27±2	−21±2	−30±1	−39±1
ΔH^≠^ (kcal mol^−1^)	6.9±0.4	10.2±0.3	6.7±0.3	5.4±0.6	6.5±0.3	6.7±0.3	4.7±0.2	2.4±0.2
ΔG^≠^ (kcal mol^−1^)	13.2±0.3	13.2±0.1	14.4±1.5	14.4±2.5	14.6±1.6	14.7±1.8	13.8±0.1	13.9±0.2

[a]–[c] are from references;[^7^, ^20^] MpDHFR denotes DHFR from *Moritella profunda*.

Previous interpretations of dynamic coupling in enzymatic reactions have focused on the temperature dependence of substrate KIEs using models that either emphasize tunnelling[^1c^, ^22^] or the relevance of transition state theory.[^1a^, ^2c^, ^23^] On the other hand, the study of enzyme KIEs represents a simplified approach to understand how protein motions couple to the chemical step on the basis that a non‐unity value of enzyme KIE indicates the coupling of protein dynamics to the chemical coordinate. Hence, our characterization of the extremely fast reaction kinetics and the transition state of the HsDHFR catalyzed reaction reveal that, contrary to a previous proposal, “protein promoting vibrations” do not facilitate the chemical step of the HsDHFR catalyzed reaction;^[1b]^ however, the results reported here show that unfavorable dynamic coupling that enhances dynamic recrossing of the activation barrier has been minimized during evolution.[^2c^, ^7^] This conclusion is also supported by a recent study on alcohol dehydrogenase from *Bacillu*s *stearothermophilus*, where the use of non‐natural and slow substrates lead to enhanced dynamic coupling.^[24]^ Our findings align with an enzymatic model where protein motions play a role in establishing a reaction‐ready configuration while chemical transformations occur in relatively static electrostatic environment. These observations offer an opportunity to probe the kinetics of mammalian DHFRs with new inhibitors that can induce anticatalytic dynamics in the transition state with potential for drug discovery.

## Conflict of interest

The authors declare no conflict of interest.

## Supporting information

As a service to our authors and readers, this journal provides supporting information supplied by the authors. Such materials are peer reviewed and may be re‐organized for online delivery, but are not copy‐edited or typeset. Technical support issues arising from supporting information (other than missing files) should be addressed to the authors.

Supplementary
