# Supplementary material for: Cryo‐kinetics Reveal Dynamic Effects on the Chemistry of Human Dihydrofolate Reductase
Source: Chembiochem. 2021 May 4;22(14):2410–4. doi: 10.1002/cbic.202100017 (PMC8360168; doi:10.1002/cbic.202100017)
Supplement: Supplementary file 1 — Supplementary [file CBIC-22-2410-s001.pdf]

ChemBioChem

Supporting Information

Cryo-kinetics Reveal Dynamic Effects on the Chemistry of Human Dihydrofolate Reductase

Aduragbemi S. Adesina, Louis Y. P. Luk, and Rudolf K. Allemann*

Table of Contents

Table of Contents 2
1.0 Experimental Procedures 3
1.1 Protein Production and Purification 3
1.2 Steady State kinetic measurements 3
1.3 Pre-Steady State kinetic measurements 3
1.3.1 Hydride transfer rate constant measured under pre-steady-state conditions 4
1.3.2 Steady-state rate constants under different conditions 6
1.4 Mass spectra of light and heavy HsDHFR 6
1.4.1 Natural abundance 'light' HsDHFR 6
1.4.2. ^{13}C, ^{15}N isotope-labeled 'heavy' HsDHFR 7
1.5 Circular dichroism analysis 8
1.6 Rate constants of chemical step catalyzed by light and heavy enzymes. 9
1.6.1. Enzyme KIE in the absence of 30% methanol 9
1.6.2. Enzyme KIE in the presence of 30% methanol 10
1.7 Substrate KIE of heavy HsDHFR 10
1.8 Activation parameters 11
2.0 References 11
3.0 Author Contributions 11

1.0 Experimental Procedures

1.1 Protein Production and Purification

The gene coding for HsDHFR (UniProt Accession ID: P00374) was expressed in *E. coli* BL21 (DE3) cells. Cells were grown in M9 media and supplemented with 50 µg/mL kanamycin, vitamins and either natural abundance or isotopically labeled ammonium chloride and glucose (that is, $^{14}NH_4Cl$/$^{15}NH_4Cl$ and ^{12}C-glucose/^{13}C-glucose) for light and heavy enzyme production, respectively. Gene expression was induced at O.D. 600 nm of 0.9 by the addition of 1 mM IPTG. The culture was further grown at 25 °C overnight. Cells were harvested by centrifugation. HsDHFR was purified using a modified protocol involving Q-Sepharose and size exclusion chromatography. The purification protocol, to be published separately by the authors, neither employ folic acid to enhance the enzyme's stability nor require a refolding step to remove protein and ligand impurities as recommended in a previous protocol. [1] Protein concentration was determined using a bicinchoninic acid assay.[2] Enzyme purity was assessed by LC/MS and circular dichroism employed to ascertain the secondary structure of the enzymes in different buffer systems.

1.2 Steady State kinetic measurements

Steady-state hydride (k_{cat}^{H}) and deuteride (k_{cat}^{D}) transfer rate constants were measured with a Shimadzu UV-spectrophotometer in MTEK buffer (50 mM MES, 25 mM Tris, 25mM ethanolamine and 150 mM KCl) with 5 mM DTT at pH 10.0 and a final enzyme concentration of 20 nM. Steady-state parameters (k_{cat} and K_M) were measured in 50 mM potassium phosphate, 150 mM KCl, 5 mM DTT pH 7.0 with the final enzyme concentration of 5 nM similar to previous reports.[3,4]

1.3 Pre-Steady State kinetic measurements

TgK Scientific stopped-flow equipment was modified for cryogenic measurements by diverting the water bath cycler from the sample handling unit to the T-pod to prevent cryo-damage of the unit. A constant flow of nitrogen ensured the optics are free from condensation. Injection volume was reduced to 80 µL and a 60 sec delay between each acquirement was used to ensure that the reacting solutions are equilibrated to the temperature within the cell block. Hydride transfer reaction by HsDHFR is fast and often complete within 0.1 sec. 20 µM enzyme was pre-incubated with 10 µM NADPH/D and the reaction initiated by the addition of 200 µM DHF. Following excitation at 297 nm, emission was measured using an output filter with a 400 nm cut-off. Hydride transfer rates were determined by fitting the relaxation of the fluorescence energy transfer from the enzyme to the reduced cofactor to a first-order exponential (Section 1.3.1). Final assay conditions were 10 µM HsDHFR, 5 µM NADPH/D and 100 µM DHF. The buffer was 50 mM potassium phosphate, 20 mM boric acid (pH, 8.5), 150 mM KCl, 5 mM DTT in the presence of 30% methanol. Pre-steady state measurements were also carried out in the absence of methanol between 0 and +20 °C using NADPD as the cofactor. NADPH/D exhibit biphasic binding to HsDHFR, especially at non-saturating concentrations.[5,6] Hence, a change in time course during fluorescence relaxation that became more prominent at lower temperatures was observed after 100 msec.

Table S1. Temperature-dependence of the steady-state kinetic parameters for the reaction of NADPH/D and DHF in MTEK buffer, pH 10.0.

**Temperature (℃)**	k_{cat}^H (s^{-1})	k_{cat}^D (s^{-1})	k_{cat}^H/k_{cat}^D
5.0	0 38± 0.01	0.19 ±0.02	2.0 ± 0.21
10.0	0.56 ± 0.04	0.25 ± 0.01	2.2 ± 0.19
15.0	1.06 ±0.02	0.50 ± 0.06	2.1 ± 0.12
20.0	1.58 ± 0.01	0.78 ± 0.04	2.0 ± 0.06
25.0	2.67 ± 0.13	1.22 ± 0.09	2.2 ± 0.09
30.0	4.42 ± 0.19	2.23 ± 0.04	2.0 ± 0.05
35.0	4 95± 0.08	2.60 ± 0.09	1.9 ± 0.04
E_a (kcal·mol^{-1})	15.54 ± 0.74	16.06 ± 0.78	A_H/A_D= 0.83 ± 0.04

MTEK buffer contains 50 mM MES, 25 mM Tris, 25mM ethanolamine and 150 mM KCl

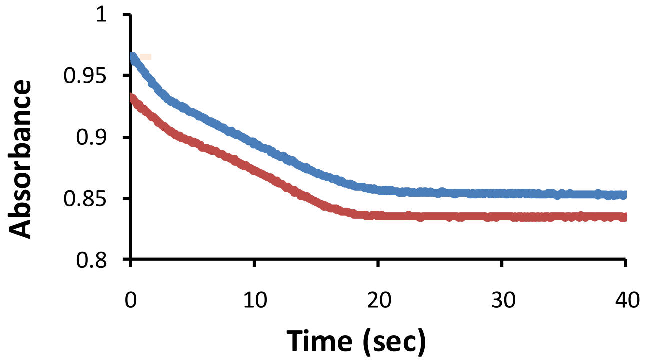

Figure S1. Multiple time-course graphs obtained from steady state measurement at 5 °C. Traces are shown as NADPH (blue) and NADPD (cherry). Identical traces were observed under different conditions at low temperatures. The initial and final rates were about seventy fold different (28.24 vs 0.38 ± 0.01 s^{-1} for k_{cat}^H).

1.3.1 Hydride transfer rate constant measured under pre-steady-state conditions

The relaxation of the fluorescence energy transfer from the enzyme to the cofactor was fitted to a single exponential with a slope function. The definition (Kinetic Studio 4.0) of the model is A exp (-R*X) + (M*X) + C, with R representing the rate constant of the best fit of the curve.

Table S2. Temperature dependence of the pre-steady state kinetic parameters for the reaction of NADPH/D and DHF catalyzed by HsDHFR in phosphate-boric-30%methanol buffer pH 8.5

Temperature (°C)	k_H (s^{-1})	k_D (s^{-1})	k_H/k_D
-20.	51.4 ± 2.4	20.8 ±3.5	2.5 ± 0.10
-15	73.4 ± 3.6	32.3 ± 1.9	2.3 ± 0.09
-10	104.9 ±3.8	45.3± 1.6	2.3 ± 0.05
-5	123.6 ± 3.9	57.6 ± 1.9	2.1 ± 0.09
0	161.7 ± 4.2	71.9 ± 3.3	2.2 ± 0.05
5	217.8 ± 6.7	94.8 ± 6.3	2.3 ± 0.05
E_a (kcal·mol^{-1})	7.8 ± 0.4	8.2 ± 0.4	A_H/A_D= 1.01 ± 0.06

Table S3. Temperature dependence of the pre-steady state kinetic parameters for the reaction of NADPD and DHF catalyzed by HsDHFR in the presence and absence of 30% methanol co-solvent.

Temperature (°C)	No solvent k_D (s^{-1})	30% methanol k_D (s^{-1})
-20	ND	20.8 ±3.5
-15	ND	32.3 ± 1.9
-10	ND	45.3 ± 1.6
-5	ND	57.6 ± 1.9
0	97.9 ± 3.1	71.9 ± 3.3
5	124.6 ± 1.9	94.8 ± 6.3
10	158.7 ± 6.5	n.d.
15	202.2 ± 6.5	n.d.
20	268.3 ± 6.1	n.d.
E_a (kcal·mol^{-1})	7.9 ± 0.2	8.2 ± 0.4

n.d. means not determined

Steady-state kinetics show that the enzyme-ligand interactions were unaffected as indicated by the K_M values (Table S5). However, minor structural effects were observed when the CD spectra were analyzed (Table S6), which might be due to the perturbation of water-molecules that had been proposed to stabilize the structure of HsDHFR.[20] In addition, the steady-state (k_{cat}) at pH 7.0 (Table S5) and pre-steady state rate constants above 10 °C were reduced due to the addition of methanol (data not shown).

Table S4. Melting temperature determined in 10 mM buffers

Enzyme and conditions	Melting temperature T_m 215 nm (°C)
Phosphate pH 7.0	37.2 ± 1.0
Boric acid pH 8.0	40.7 ± 0.2
Boric acid with 30% MeOH pH 8.0	30.3 ± 0.1

1.3.2 Steady-state rate constants under different conditions

Table S5. Steady-state parameters of HsDHFR at 20 ℃

Buffer	k_{cat} (s^{-1})	K_M DHF (μM)	K_M NADPH (μM)
MTEK, pH 10.0	1.58 ± 0.01	5.3 ± 1.9	26.0 ± 9.6
Phosphate, pH 7.0	10.43 ± 0.87	0.14 ± 0.33	3.81 ± 1.33
Phosphate with 30% methanol, pH 7.0	2.82 ± 0.86	0.14 ± 0.06	2.9 ± 0.80

1.4 Mass spectra of light and heavy HsDHFR

1.4.1 Natural abundance ‘light’ HsDHFR

Intact first methionine (major peak): Expected: 21453 Da and calculated: 21452 ± 0.87 Da

Cleaved first methionine (minor peak): Expected: 21322 Da and calculated: 21321 ± 0.67 Da

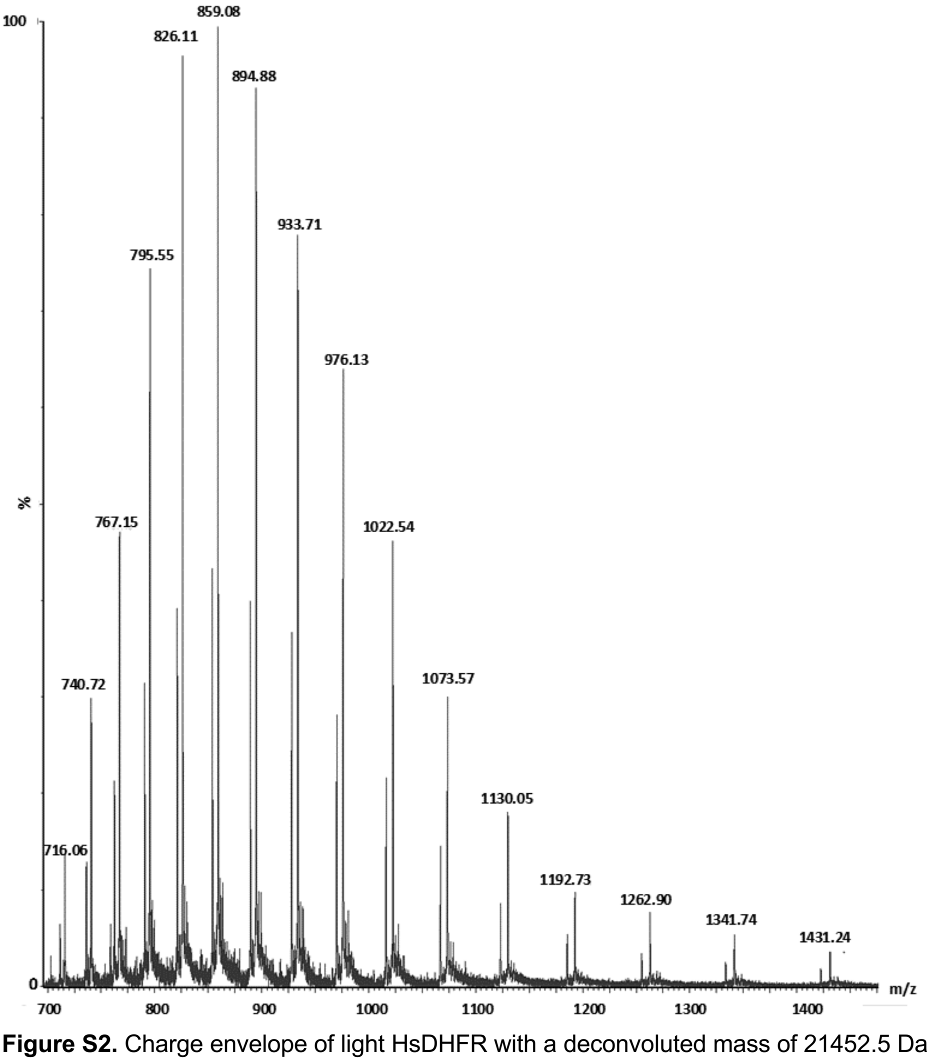

Figure S2. Charge envelope of light HsDHFR with a deconvoluted mass of 21452.5 Da

1.4.2. ^{13}C, ^{15}N isotope-labeled 'heavy' HsDHFR

Intact first methionine: Expected: 22666 Da and calculated:22605 ± 0.52 Da (99.7% heavy atom incorporation calculated from $C_{968}H_{1520}N_{254}O_{280}S_8$)

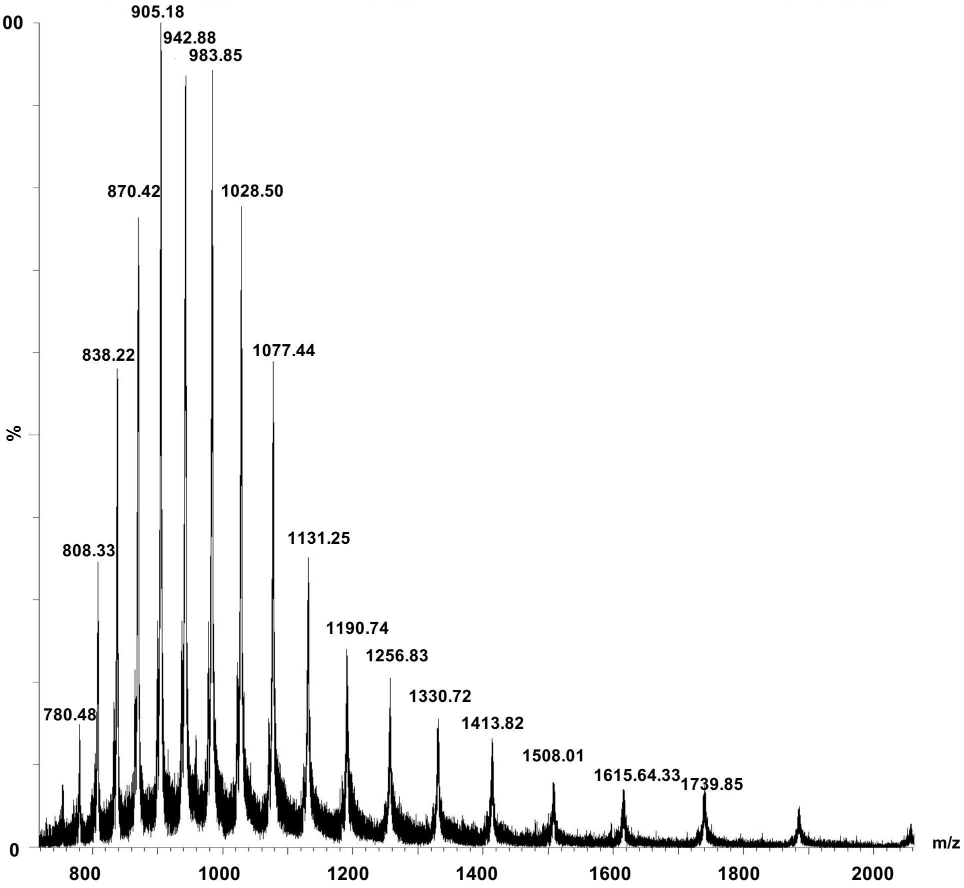

Figure S3. Charge envelop of heavy HsDHFR with a deconvoluted mass of 22605.5 Da

1.5 Circular dichroism analysis

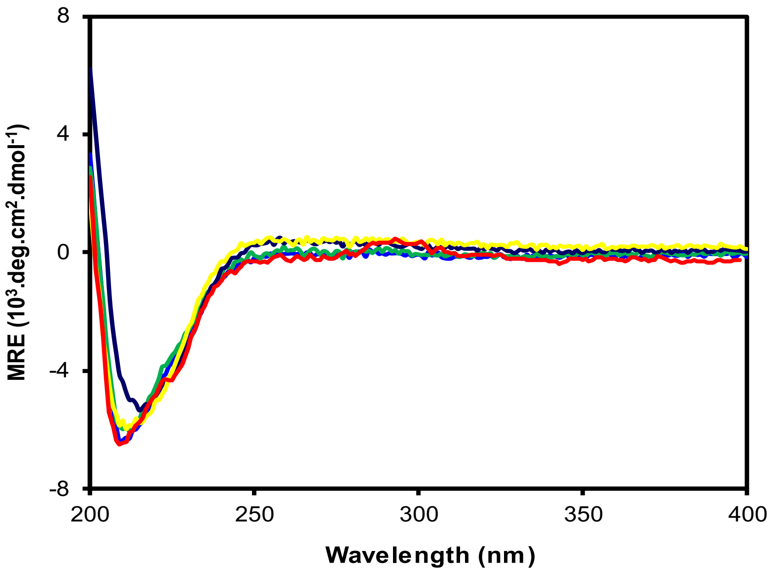

Figure S4. CD spectra at 20 ºC for HsDHFR (10 µM) in 10 mM buffers. Light enzyme in boric acid pH 8.0 (green), boric acid with 30% methanol pH 8.0 (dark blue), boric acid pH 10.0 (yellow), phosphate pH 7.0 with folate ligand (blue) and heavy enzyme in phosphate pH 7.0 with folate ligand (red)

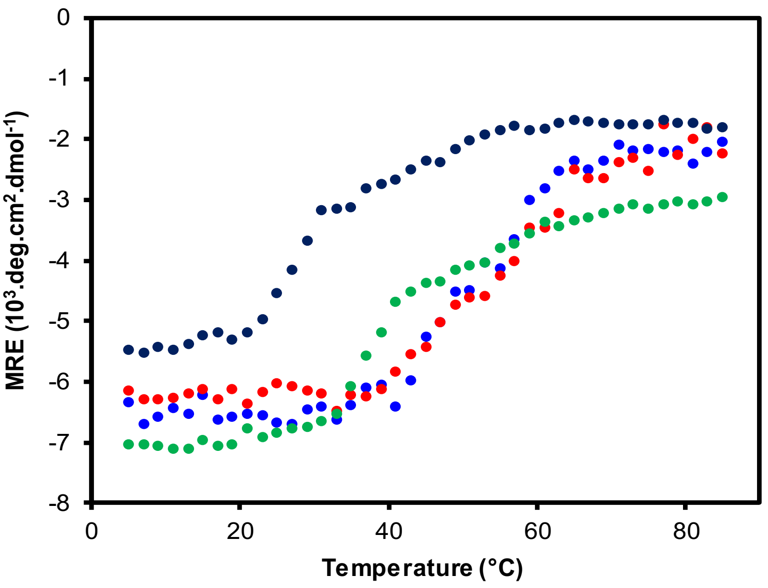

Figure S5. Melting temperature of HsDHFR (10 µM) in 10 mM buffers taken at 215 nm. Light enzyme in boric acid pH 8.0 (green), boric acid with 30% methanol pH 8.0 (dark blue), phosphate pH 7.0 with folate ligand (blue) and heavy enzyme in phosphate pH 7.0 with folate ligand (red).

Table S6. Steady-state parameters of light and heavy HsDHFR at 20 ℃

Buffer	k_{cat} (s^{-1})	K_M DHF (µM)	K_M NADPH (µM)
Light HsDHFR Phosphate, pH 7.0	10.43 ± 0.87	0.14 ± 0.33	3.81 ± 1.33
Heavy HsDHFR Phosphate, pH 7.0	10.55 ± 0.78	0.10 ± 0.02	3.91 ± 1.19

Table S7. CD melting temperature of HsDHFR in 10 mM buffer

Conditions	Melting Temperature at 215 nm (T_m) (°C)
Light enzyme in boric acid, pH 8.0	40.7 ± 0.20
Light enzyme in boric acid and 30% MeOH, pH 8.0	30.3 ± 0.10
Light enzyme with folate, phosphate pH 7.0	55.0 ± 0.20
Heavy enzyme with folate, phosphate pH 7.0	55.1 ± 0.10

1.6 Rate constants of chemical step catalyzed by light and heavy enzymes.

Table S8. Temperature dependence of the pre-steady state kinetic parameters for the reaction of NADPH and DHF catalyzed by light and heavy HsDHFR in phosphate-boric-30% methanol buffer pH 8.5

Temperature (°C)	Light k_H (s^{-1})	Heavy k_H (s^{-1})	Enzyme KIE
-20.0	51.4 ± 2.4	29.9 ±2.4	1.7 ± 0.15
-17.5	70.8 ± 3.8	40.4 ± 1.8	1.8 ± 0.12
-15.0	73.4 ± 3.6	43.8 ± 1.3	1.68 ± 0.09
-10.0	104.9 ±3.7	74.3 ± 4.0	1.44 ± 0.05
-5.0	123.6 ± 3.9	102.3 ± 8.5	1.21 ± 0.08
0.0	161.7 ± 4.2	154.0 ± 6.3	1.05 ± 0.06
5.0	217.8 ± 6.7	203.0 ± 4.3	1.07 ± 0.05
E_a (kcal·mol^{-1})	7.5 ± 0.40	10.7 ± 0.32	

1.6.1. Enzyme KIE in the absence of 30% methanol

Table S9. Pre-steady state rate constant for deuteride transfer catalyzed light and heavy HsDHFR in the absence of cosolvent

Temperature (°C)	Light k_D (s^{-1})	Heavy k_D (s^{-1})	Enzyme KIE
0	97.9 ± 3.1	100.7 ±1.8	0.97 ± 0.04
20	268.3 ± 6.1	282.9 ± 18.0	0.95 ± 0.07
E_a (kcal·mol^{-1})	8.01 ± 0.20	8.20 0.44	

1.6.2. Enzyme KIE in the presence of 30% methanol

Table S10. Pre-steady state rate constant for the deuteride transfer catalyzed by light and heavy HsDHFR in 30% methanol buffer

Temperature (°C)	Light k_D (s^{-1})	Heavy k_D (s^{-1})	Enzyme KIE
-15	32.3 ± 1.9	17.8 ±1.6	1.81 ± 0.11
5	94.8 ± 6.3	89. ± 8.3	1.06 ± 0.11
E_a (kcal·mol^{-1})	7.9 ± 0.2	11.6 ± 0.7	

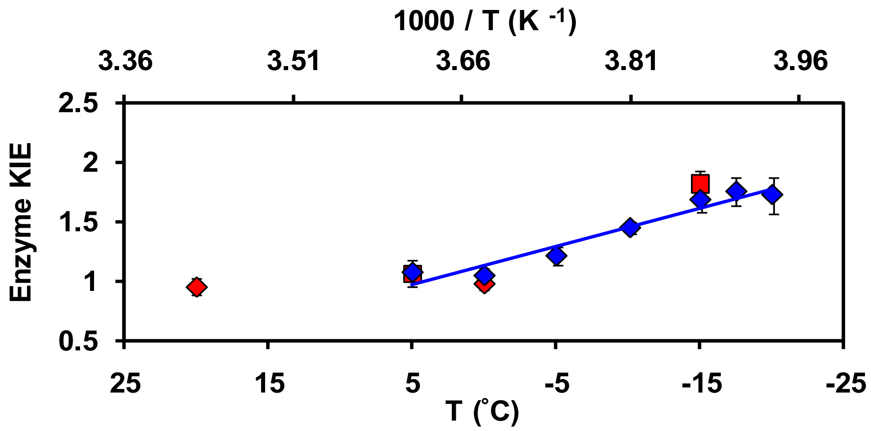

Figure S6. Temperature-dependence of enzyme KIE for the transfer of hydride (◆) and deuteride in buffers with (■) and without 30% methanol (◆).

1.7 Substrate KIE of heavy HsDHFR

Table S11. Pre-steady state rate constant hydride and deuteride transfer catalyzed by heavy HsDHFR in 30% methanol buffer

Temperature (°C)	Heavy k_H (s^{-1})	Heavy k_D (s^{-1})	(k_H/k_D)
-15	43.9 ± 1.3	17.8 ±1.6	2.5 ± 0.09
5	203.0 ± 4.3	89.1 ± 8.3	2.3 ± 0.10
E_a (kcal·mol^{-1})	10.5 ± 0.5	11.6 ± 0.7	

1.8 Activation parameters

Activation parameters were calculated from a plot of the linearized Eyring equation $In\ \frac{k}{T} = \frac{-\Delta H^{\ddagger}}{R} . \frac{1}{T} + In\ \frac{k_B}{h} + \frac{\Delta S^{\ddagger}}{R}$, where k_B is the gas constant, *h* is Plank's constant, $\Delta H^{\ddagger}$ is the enthalpy of activation and $\Delta S^{\ddagger}$ is the entropy of activation. The values fo the activation parameters were determined from kinetic data obtained from a plot of $In\ \frac{k}{T}$ vs $\frac{1}{T}$. The equation is a straight line with negative slope, $\frac{-\Delta H^{\ddagger}}{R}$ and a y-intercept $In\ \frac{k}{T} + \frac{\Delta S^{\ddagger}}{T}$. The slope and intercepts were obtained from a linear regression fit using StatFi package plugin on Microsoft Excel. Free energy of activation was calculated using equation $\Delta G^{\ddagger} = \Delta H^{\ddagger} - T\Delta S^{\ddagger}$ and the respective temperatures reported in the manuscript.

2.0 References

(1) *FEBS Lett.* **2011**, *585* (22), 3528–3532.

(2) *J. Am. Chem. Soc.* **2003**, *125* (44), 13372–13373.

(4) *Biochemistry* **2004**, *43* (14), 4119–4127.

(5) *J. Biol. Chem.* **1990**, *265* (5), 2740–2748.

(6) *J. Biol. Chem.* **1989**, *264* (5), 2625–2633.

3.0 Author Contributions

R.K.A. conceived the original idea. A.S.A. performed the experiments and analysed the data. R.K.A, L.Y.L and A.S.A interpreted the results. A.S.A, L.Y.L. and R.K.A. wrote the manuscript.
